# Electrocardiogram: the saviour for this patient

**DOI:** 10.1007/s12471-015-0794-2

**Published:** 2015-12-08

**Authors:** A. Chawla, S. Swarup, V. Gaikwad

**Affiliations:** 1 Department of Diagnostic Radiology, Khoo Teck Puat Hospital, 90 Yishun Central, 768828 Yishun, Singapore; 2 Department of Accident and Emergency, Khoo Teck Puat Hospital, Yishun, Singapore

The ECG shows a regular rhythm with mildly elevated ST segment in the precordial leads. There is a biphasic T wave in V1 and V2 leads suspicious for Wellens syndrome. The patient refused an invasive procedure, hence a coronary CT angiography was performed that showed severe stenosis in the proximal left anterior descending artery (LAD) (Fig. 1). Subsequently, he agreed to catheter angiography that confirmed the CT findings (Fig. 2). The lesion was treated by placement of a stent. The Wellens ECG is an ominous sign of severe proximal LAD stenosis that can cause a large anterior wall myocardial infarct if left untreated [1]. Hence it is imperative that the emergency physician is aware of this pattern of ECG to prevent myocardial damage by promptly ordering a cardiac catheterisation.

**Fig. 1:**
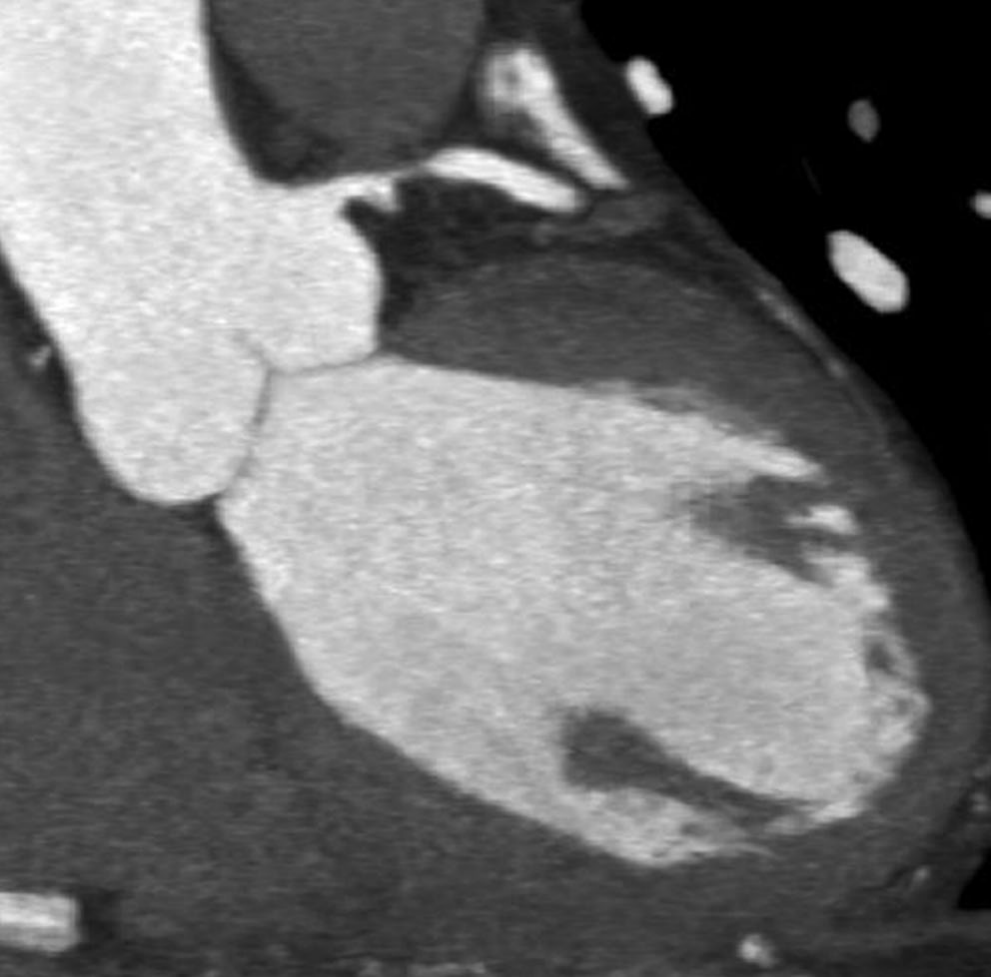
Thick maximum intensity projection coronary CT angiography image

**Fig. 2:**
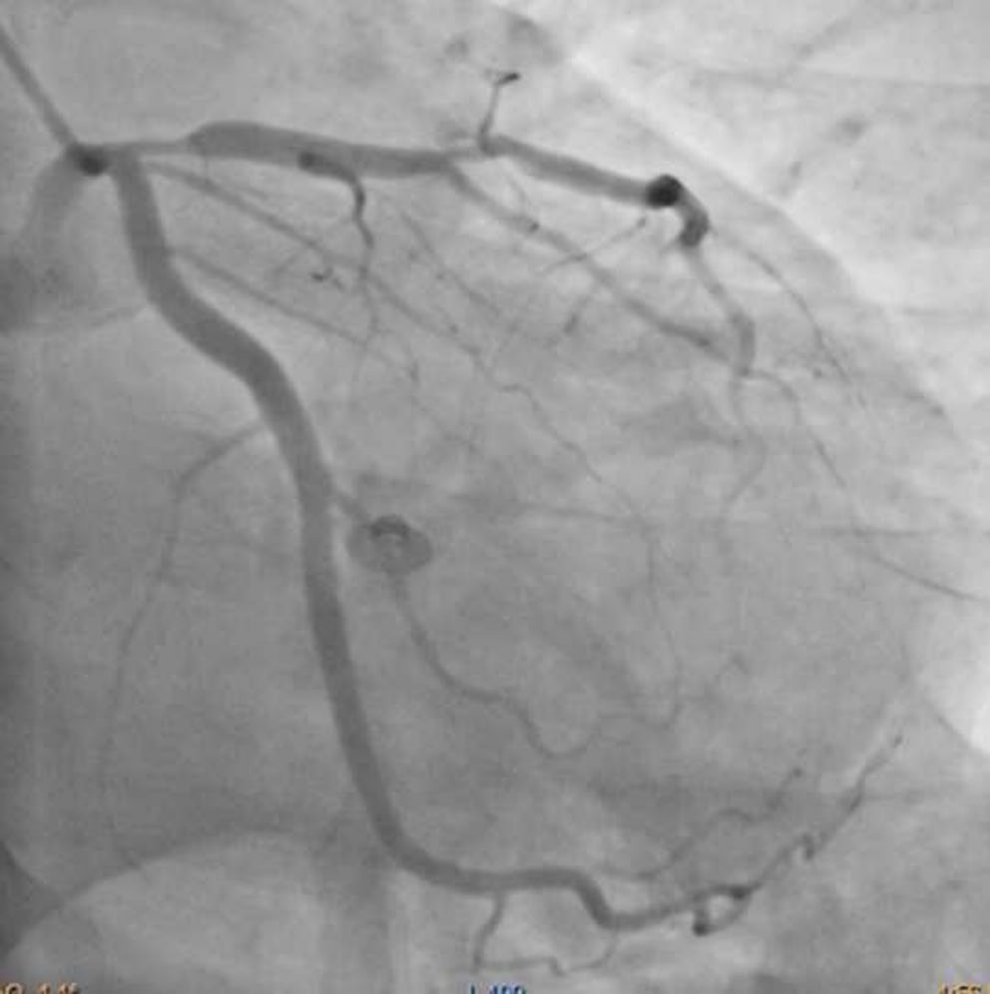
Catheter angiography with left main coronary artery injection

## Conflict of interest

The authors have no conflict of interest related to this report.
